# A forest‐specialist carnivore in the middle of the desert?Comments on https://onlinelibrary.wiley.com/doi/full/10.1002/ece3.5230

**DOI:** 10.1002/ece3.6132

**Published:** 2020-03-25

**Authors:** Darío Moreira‐Arce, Eduardo A. Silva‐Rodríguez, Constanza Napolitano, Guillermo D’Elía, Javier Cabello, Javier Millán, Ezequiel Hidalgo‐Hermoso, Ariel A. Farías

**Affiliations:** ^1^ Laboratorio de Estudios del Antropoceno Facultad de Ciencias Forestales Universidad de Concepción Concepción Chile; ^2^ Facultad de Ciencias Forestales y Recursos Naturales Instituto de Conservación, Biodiversidad y Territorio Universidad Austral de Chile Valdivia Chile; ^3^ Departamento de Ciencias Biológicas y Biodiversidad Universidad de Los Lagos Osorno Chile; ^4^ Instituto de Ecología y Biodiversidad (IEB) Santiago Chile; ^5^ Facultad de Ciencias Instituto de Ciencias Ambientales y Evolutivas Universidad Austral de Chile Valdivia Chile; ^6^ Facultad de Medicina Veterinaria Universidad San Sebastián Puerto Montt Chile; ^7^ Facultad de Ciencias de la Vida Universidad Andrés Bello Santiago Chile; ^8^ Instituto Agroalimentario de Aragón‐IA2 Universidad de Zaragoza‐CITA Zaragoza Spain; ^9^ Fundación ARAID Zaragoza Spain; ^10^ Departamento de Conservación Parque Zoologico Buin Zoo Buin Chile; ^11^ Departamento de Ecología y Gestión Ambiental Centro Universitario Regional Este (CURE‐Maldonado) Universidad de la República Maldonado Uruguay; ^12^ Center of Applied Ecology and Sustainability (CAPES) Pontificia Universidad Católica de Chile Santiago Chile; ^13^ Centro de Investigación e Innovación para el Cambio Climático (CIICC) Universidad Santo Tomás Santiago Chile

**Keywords:** genus *Lycalopex*, geographic distribution, lack of evidence, research design

## Abstract

We present comments on an article recently published in Ecology and Evolution (“High‐resolution melting of the cytochrome B gene in fecal DNA: A powerful approach for fox species identification of the *Lycalopex* genus in Chile”) by Anabalon *et al.* that reported the presence of Darwin's fox (*Lycalopex fulvipes*), a temperate forest specialist, in the hyperarid Atacama Desert of northern Chile. We argue that this putative record lacks ecological support in light of ongoing research on this endangered species, and contains numerous methodological flaws and omissions related to the molecular identification of the species. Based on these issues, we suggest the scientific community and conservation decision‐makers disregard the alleged presence of the Darwin's fox in the Atacama Desert.

## Abstract 2

We present a Reply letter for a recent paper reporting the presence of Darwin´s fox, a forest‐specialist canid, in the Atacama Desert, 1,200 km north of its known distribution.

The ongoing vertebrate population declines reported in the Living Planet Report (WWF, 2018) challenges conservationists and environmental agencies to provide reliable information, particularly on endangered species, to inform decisions and help to achieve conservation goals. The discovery of new populations is particularly important for conservation planning, especially when laying outside the known geographic distributions (Guisan et al., 2013; Margules & Pressey, 2000). In a recent issue of Ecology and Evolution, Anabalón et al. (2019) used high‐resolution melting (HRM), based on thermal denaturation of DNA amplicons, as a method to identify different Chilean fox species (*Lycalopex* spp.) from scat samples collected in the Atacama Desert, northern Chile (ca. 26°31′S, 70°30′W; Figure 1), one of the most arid environments in the world (McKay et al., 2003). The study reports a remarkable discovery: the presence of the endangered Darwin's fox (*Lycalopex fulvipes*), in the hyperarid Atacama Desert, more than 1,200 km further north than the limit of its currently known distribution (Figure 1). We argue this record is questionable in light of current understanding on the ecology of the species and, based on the information provided by Anabalon et al., we question the reliability of its molecular identification.

**Figure 1:**
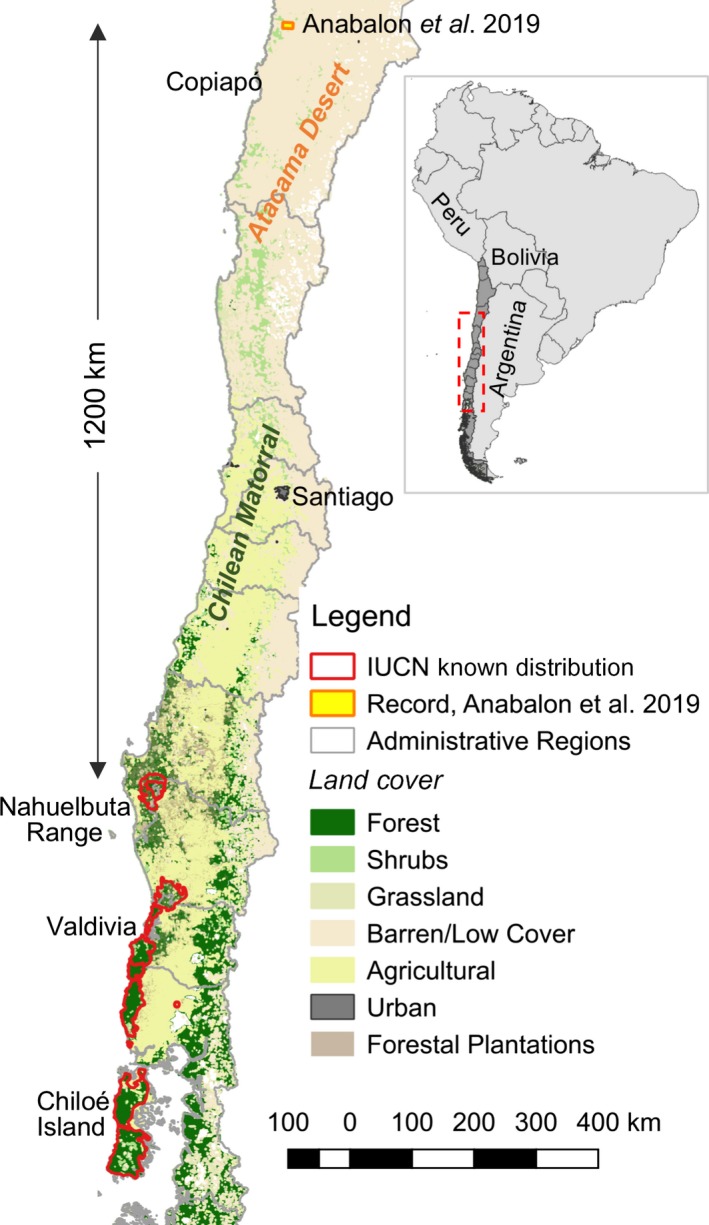
Known distributional range of Darwin's fox based on the IUCN assessment (Silva‐Rodríguez et al., 2016) and the new putative record reported in Anabalón et al. (2019). Vegetation cover simplified to coarser categories from Land Use—Chile digital map available at http://datos.cedeus.cl/layers/geonode:cl_uso_suelo_geo

Several new records of the Darwin's fox have been reported in recent years (D'Elía, Ortloff, Sanchez, Guinez, & Varas, 2013; Farias et al., 2014; Jiménez, 2019; Silva‐Rodríguez et al., 2018; Vilà et al., 2004). These findings have been able to fill the gaps between previously known populations (Silva‐Rodríguez et al., 2016). In addition, all published records limit the species range to within the temperate and humid Valdivian rainforest. Indeed, diverse studies have shown that the species is consistently associated with native forest (Farias & Jaksic, 2011; Jiménez, Marquet, Medel, & Jaksic, 1991; Moreira‐Arce, Vergara, & Boutin, 2015; Silva‐Rodríguez et al., 2018) and specifically to areas with high understory cover (Moreira‐Arce et al., 2016), although other nearby ecosystems—such as beaches and dunes near to primary forest—can be used (Jiménez, 2007). All of these studies are congruent in showing that the Darwin's fox is a forest‐specialist species and therefore casts doubts on its occurrence in one of the driest deserts of the world.

If confirmed, such novel finding would be remarkable and would radically change our understanding of the natural history of this endangered canid. However, after a careful reading of the paper by Anabalon et al. we identified a number of issues that cast doubts on the putative new record. Problems include the inadequate usage of available knowledge on Chilean canids and the plausible explanations for the putative new record of Darwin's fox, aspects of study design, the lack of reported controls and validation analyses, and failure to consider basic aspects of molecular‐based organism identification.

Anabalon et al. invest little of the manuscript on the ecology of the Darwin's fox. Specifically, the paper fails to mention that the species is a forest specialist. We find this remarkable given that the putative new record is from a desert. In fact, large sections of relevant literature on the species are omitted. We also note that in the three paragraphs devoted to discussing the putative new record, authors only cited a single article (Medel & Jaksic, 1988) on *Lycalopex* biology, published three decades ago (i.e., well before the large majority of the studies on the Darwin's fox were published). As such, the findings of Anabalon et al. are not presented alongside the contemporary understanding of Chilean fox species ecology. In addition, other mistakes are made. For instance, the authors contend that the South American gray fox (*L. griseus*) is present on Chiloe Island (a stronghold for the Darwin's fox); this is incorrect (see González del Solar & Rau, 2004). We also identified caveats on the study design, omissions to mention (if conducted) laboratory controls directed to validate such a remarkably finding, and lack of considerations of alternative scenarios that might be more biologically likely.

The findings of Anabalon et al. are centered on the molecular identification of three species of fox found in Chile (namely, the culpeo fox *L. culpaeus*, the South American gray fox, and the Darwin's fox). We question why the authors did not also include the pampas fox (*L. gymnocercus*) and Sechuran fox (*L. sechurae*) as these species occur in arid and semi‐arid environments in nearby countries (Cossíos, 2010; Lucherini & Luengos Vidal, 2008). Instead, the authors only included sequences of the Chilean species of *Lycalopex* as a reference to match sequences recovered from unknown fecal samples collected in the Atacama Desert. Here, a single sequence per species was used to establish the standard melting temperature of each species. We consider this reference dataset to be insufficient, as it does not take into consideration intraspecific variation. Most studies applying HRM use four to 10 different samples per species to generate a database of Tm and curve profiles that encompass potential intraspecific variation (Mandviwala, Shinde, Kalra, Sobel, & Akins, 2010; Peña et al., 2012). An increased sample size helps to account for intraspecific variation and validates the possible melting curves and temperatures for each reference species. In addition, each sample needs to be replicated, comparing HRM profiles to their corresponding DNA sequences, to discard, among other issues, cases of contamination (highly likely when working with DNA extracted from nonconventional samples; see Peña et al., 2012; Waits & Paetkau, 2005). Besides, Anabalon et al. did not report whether negative controls were used to discard cases of cross‐contamination among samples, and none of these validation steps are described.

In addition, the authors failed to follow well‐established practices in studies of species identification (e.g., Farrell, Roman, & Sunquist, 2000; Johnson et al., 1998; Napolitano et al., 2008; Vilà et al., 2004). No information is provided on the specimens used to gather the reference sequences or the collections that house them. Similarly, no locality data for the reference specimens are given. These are not trivial omissions and combine to make the method of Anabalon et al. unrepeatable and the derived results weak.

Although the genetic differentiation among *Lycalopex* species are not fully understood, the available literature suggests that their variation is geographically structured (D'Elía et al., 2013; Tchaicka et al., 2016). For example, the northern Chilean population of culpeo fox has both genetic (Yahnke et al., 1996) and morphological differences respect to those of central and southern Chile, which have led some authors to hypothesize that both forms correspond to different subspecies (Guzmán, D'Elía, & Ortiz, 2009). Moreover, mitochondrial variants of the culpeo fox form a paraphyletic group with respect to those of the South American gray fox (Yahnke et al., 1996). As such, using a single reference sequence per species could be insufficient as mitochondrial variants of one species could be more similar to variants of other species (see below). As the reference sequence of *L. fulvipes* must be from its currently known distribution in southern Chile, one would expect to find that two populations separated by at least 1,200 km (if the reference comes from the Nahuelbuta Range, Figure 1) could have some mutations of difference in their DNA sequence (divergence), thus yielding different observable Tms (see Napolitano et al., 2014 for the number of substitutions differences in the mtDNA among populations of another carnivore species). Authors state that the Tm values “did not vary within a species, hence, there were no deviations in Tm detectable,” but—despite being nearly unreadable—figure 1b in Anabalón et al. (2019) suggests there is variation in the Tm values from the fecal samples, and thus, we cannot identify three distinct Tm clusters. In fact, the putative Darwin's fox fecal sample from the desert is not identifiable in figure 1b nor figure 1d, unless it was identical to the reference sample. This pattern would also emerge in a scenario of cross‐contamination from the reference to the fecal sample.

Since HRM analysis relies on subtle differences in the shape of melt curves of amplicons, it is important to ascertain in advance whether the studied genetic segment has sufficient diversity among species, while being conserved within the species, to allow their discrimination by HRM analysis (Mandviwala et al., 2010). Anabalon et al. do not report the number of variable sites in the 200 bp fragment analyzed. They illustrate their work (see figure 1 in Anabalon et al.) with partial electropherograms (presumably the ones gathered by them) of cytb fragments of the targeted species; the bases of ca. 80 sites can be read from the figure; in that fragment, each species pair differs in a single site (ca. 1.25%). As said above, the consistency of these differences when large population samples are analyzed is unknown. The small uncovered variation translates into small differences in Tms among species (only 0.2 to 0.5ºC difference), which may be likely prone to wrong inferences when not properly validated using an adequate panel of haplotypic variants of the targeted species. Indeed, a low level of variation in the targeted DNA segment could hamper the resolution at the species level (Mandviwala et al., 2010).

Remarkably, given this is the first usage of HRM to identify any species of *Lycalopex*, it is of interest that the putative sequence of Darwin's fox from Atacama, as well some of the others belonging to the other two species, be sequenced to confirm species identity, which was not the case. A multiple sequence alignment comparing reference sequences along with those to be identified at the species level, and others downloaded from GenBank, would have helped to evaluate the results obtained. It appears that no such control analysis was conducted. Instead, the only DNA sequence they claim to have deposited in GenBank (GenBank: AF028151) corresponds to a sequence gathered in the study of Wayne et al. (1997). This means we were not able to properly check sequence identity. However, in a final attempt to clarify this issue, we gathered a fragment of 79 bp of the electrochromatograms shown by Anabalon et al. in their figure 1 and compared them with sequences of *Lycalopex* species downloaded from GenBank (after a sequence alignment done with CLUSTAL W, as implemented in MEGA 6; Tamura, Stecher, Peterson, Filipski, & Kumar, 2013). Results (p‐distance calculated in MEGA 6) showed that the putative sequence of *L. culpaeus* has a 100% match with sequences of *L. vetulus* (GenBank: AF028148) and *L. culpaeus* (GenBank: AF028151). The putative sequence of *L. fulvipes* has the largest similarity (98.7%) with the two mentioned sequences and the putative sequence of *L. culpaeus*; we note that cyt b sequences of *L. fulvipes* were not available in GenBank. Finally, the putative sequence of *L. griseus* has the highest identity (98.7%) with a sequence of *L. griseus* (GenBank: AF028152).

Even if the result suggesting that Darwin's fox inhabit Atacama was not spurious (i.e., not affected by sample contamination, an inadvertent mixture of samples, or derived from an analysis with low resolutive power), there are some alternative scenarios, which we consider to be biologically more likely that were not considered by Anabalon et al. It is an established fact that species are not always monophyletic at a given locus (Avise & Ball, 1990); this potential incongruence between a gene tree and the species tree can be caused by well‐understood biological processes. One possibility is introgression (in this case of the *L. fulvipes* mtDNA into populations of *L. griseus* or *L. culpaeus*) after a hybridization event (a process well known in several mammal species, including canids, e.g., Lehman et al., 1991). We lack evidence to suggest that the putative Darwin's fox‐like cytb haplotype collected in Atacama was recovered from the feces of a culpeo or a South American gray fox introgressed with Darwin's fox mitochondrial genome; but certainly, it is noteworthy that this possibility was dismissed by Anabalon et al. when they state “There are no reports on hybridization between species of the *Lycalopex* genus, only observations and stories of local people in the field…”. We argue this hypothesis should not be ruled out in view of evidence of potential hybridization processes in *Lycalopex* foxes (Silva, 2015; Tchaicka et al., 2016). In addition, incomplete sorting of ancestral polymorphisms, a fairly common process, is a major source of incongruence between gene trees and species trees (Pamilo & Nei, 1988). Therefore, it is possible that some mitochondrial variants existing in culpeo or South American gray fox populations are more closely related (and hence, more similar) to variants of Darwin's fox than to other variants of its own species. As the genetic variation of the three species is far from been adequately characterized, this possibility cannot be ruled out. Anabalon et al. do not mention this possibility despite it being a common consideration in comparable studies (e.g., Kutschera et al., 2014; Pagès et al., 2013). The assessment of the variation at the nuclear genome would allow the testing of these scenarios; such suggestion was not advanced by Anabalon et al. that took *prima facie* their mitochondrial based results. Finally, although the authors stated that they conducted camera‐trapping to monitor the presence of target fox species, results of this effort were not reported. This information might have provided valuable support for the results presented, especially in the case of the Darwin's fox, which is phenotypically distinct.

It is interesting that authors stated “Surprisingly, we could detect one *L. fulvipes* sample, which was not expected to be present in our study area,” and instead of critically evaluating if this (clearly surprising) finding was the result of a methodological or interpretation error (see above), they hypothesized that “a possible cause for migrations into new habitats could be a response to climate change or events such as forest fires.” There is no way to support such statements on either empirical or theoretical grounds. First, a recent niche modeling study (not cited by Anabalon et al.) predicts that under two climate scenarios, the distribution range of Darwin's fox may move south of its current known distribution range (Molina, Castillo, & Samaniego, 2018), toward the southern fraction of the Valdivian forest and northern portion of Magellanic rain forest, but never toward hyperarid northern ecosystems as suggested by Anabalon et al. Second, the extent of recent fire activity, including large forest fires that occurred during 2016–2017, only slightly overlapped the northern distribution range of the Darwin's fox, affecting mainly human‐modified landscapes (McWethy et al., 2018), which are unsuitable habitats for Darwin's fox (Moreira‐Arce et al., 2015; but see Moreira‐Arce et al., 2016 for exotic plantations). In consequence, the large (i.e., ca. 1,200 km) dispersal of foxes toward northern Chile across unsuitable ecosystems (Escobar, Qiao, Cabello, & Peterson, 2018; Molina et al., 2018) implied by Anabalon et al. makes no biological sense in light of the natural history of this species.

The Darwin's fox is an endangered species (Silva‐Rodríguez et al., 2016), and governmental agencies are currently working in the development of its conservation plan. In this context, a new record would be welcome news. Unfortunately, when the numerous issues we have outlined are considered together, they call into question the credibility of Anabalon et al.’s paper. We consider that the evidence strongly suggests the alleged detection of Darwin's fox in the Atacama Desert is a consequence of a mistake in the laboratory or of the interpretation of a deficiently designed study. As such, we suggest that the scientific community and conservation decision‐makers disregard the alleged presence of the Darwin's fox in the Atacama Desert. That said, we salute any initiatives that aim to investigate the ecology of Chilean foxes as they remain poorly understood on several basic aspects.

## CONFLICT OF INTEREST

None declared.

## AUTHOR CONTRIBUTIONS

DMA and ESR drafted the first version of this manuscript. All authors contributed to draft versions of it.
